# Fluorescent nucleic acid probe in droplets for bacterial sorting (FNAP-sort) as a high-throughput screening method for environmental bacteria with various growth rates

**DOI:** 10.1371/journal.pone.0214533

**Published:** 2019-04-17

**Authors:** Yuri Ota, Kanako Saito, Taeko Takagi, Satoko Matsukura, Masamune Morita, Satoshi Tsuneda, Naohiro Noda

**Affiliations:** 1 Department of Life Science and Medical Bioscience, Waseda University, Tokyo, Japan; 2 Biomedical Research Institute, National Institute of Advanced Industrial Science and Technology (AIST), Ibaraki, Japan; Universite Paris-Sud, FRANCE

## Abstract

We have developed a new method for selectively sorting droplets containing growing bacteria using a fluorescence resonance energy transfer (FRET)-based RNA probe. Bacteria and the FRET-based RNA probe are encapsulated into nanoliter-scale droplets, which are incubated to allow for cell growth. The FRET-based RNA probe is cleaved by RNase derived from the bacteria propagated in the droplets, resulting in an increase in fluorescence intensity. The fluorescent droplets containing growing bacteria are distinguishable from quenching droplets, which contain no cells. We named this method FNAP-sort based on the use of a fluorescent nucleic acid probe in droplets for bacterial sorting. Droplets containing the FRET-based RNA probe and four species of pure cultures, which grew in the droplets, were selectively enriched on the basis of fluorescence emission. Furthermore, fluorescent droplets were sorted from more than 500,000 droplets generated using environmental soil bacteria and the FRET-based RNA probe on days 1, 3, and 7 with repeated incubation and sorting. The bacterial compositions of sorted droplets differed on days 1, 3, and 7; moreover, on day 7, the bacterial composition of the fluorescent droplets was drastically different from that of the quenching droplets. We believe that FNAP-sort is useful for high-throughput cultivation and sorting of environmental samples containing bacteria with various growth rates, including slow-growing microbes that require long incubation times.

## Introduction

Droplet microfluidics-based systems have exhibited the capacity for extremely high-throughput assays [1–3]. In such systems, each aqueous droplet suspended in carrier oil through a water-in-oil emulsion process is available as an independent femto-, pico-, or nanoliter volume reactor. Uniform droplets are generated at a rate of thousands per second using a microfluidic device. Droplet reactors possess significant advantages, such as miniaturization, compartmentalization, and parallelization [4, 5]. Droplet microfluidics-based systems have thus been successfully utilized in droplet digital PCR [6, 7], high-throughput screening applications [8–10], the analysis of enzymatic kinetics [11], and the engineering of proteins through directed evolution [12, 13].

Droplet microfluidics-based techniques have been used for the cultivation of representative model bacteria, such as *Escherichia coli*, *Bacillus subtilis*, and *Pseudomonas fluorescens* [14–17]. Furthermore, Liu et al. demonstrated that the rare and slow-growing species *Paenibacillus curdlanolyticus* could be isolated from a cocktail of abundant and fast-growing *E*. *coli* using a droplet microfluidics technique [18]. The compartmentalization of bacteria into droplets is an effective means of preventing competition between slow-growing and fast-growing species; thus, this technique is useful for isolating rare and slow-growing species from mixed cultures in which fast-growing species are abundant.

Despite its considerable potential, only a few droplet microfluidics-based platforms for the cultivation of environmental microorganisms have been reported to date [19, 20]. The lack of simple detection and sorting methods for droplets containing bacteria hampers the widespread use of droplet microfluidics techniques for this purpose. Zang et al. encapsulated *Streptomyces puniceus* JA2640, which has been generally used for antibiotic screening, into picoliter-scale droplets [21]. Droplets containing spores and small mycelial pellets of *S*. *puniceus* JA2640 were differentiated from empty droplets using a photodiode under bright-field illumination. Although this detection method was effective at sorting droplets containing spores and small mycelial pellets, it required high-speed imaging analysis with a complicated calculation process. In addition, the conversion of non-fluorescent resazurin into resorufin by bacterial metabolites is commonly used as a fluorescent marker to detect droplets containing growing bacteria [22–24]. Resorufin is known however to leak from droplets, and this leakage limits the incubation time to several hours [22, 25]. To overcome these limitations, it is necessary to develop a simple and reliable detection and sorting method for droplets in which bacteria are encapsulated and grown, including those containing slow-growing microbes.

Here, we developed a new method for the selective sorting of droplets containing growing bacteria from a droplet complex on the basis of fluorescence resonance energy transfer (FRET). This method has been named fluorescent nucleic acid probe in droplets for sorting bacteria (FNAP-sort). In this method, a FRET-based RNA probe, which is usually quenched by FRET, is used. A ribonuclease such as an RNase cleaves the FRET-based RNA probe, resulting in an increase in its fluorescence intensity. In FNAP-sort, the FRET-based RNA probe is encapsulated with bacterial cell(s) into droplets. As the bacteria grow in the droplets, the fluorescence intensity of the droplets increases due to the cleavage of the FRET-based RNA probe by an RNase secreted from the growing bacteria. Droplets containing growing bacteria can therefore be easily sorted on the basis of an increase in fluorescence intensity.

## Materials and methods

### Preparation of bacterial samples

We used four different types of bacteria in the present report. *E*. *coli* K12, *B*. *subtilis* NBRC13719, *Streptomyces aureofaciens* NBRC12843, and *Bradyrhizobium japonicum* NBRC14783 were used as model bacteria. *E*. *coli* from a glycerol stock was inoculated into 2 mL of lysogeny broth (LB) medium and incubated with shaking at 37°C for 16 hours. The concentration of *E*. *coli* cells was determined on the basis of the optical density at 600 nm (OD_600_), which was measured by GeneQuant 1300 (GE Healthcare, Waukesha, WI, USA). The pre-cultured *E*. *coli* was washed and resuspended in fresh LB medium. A dilution equivalent to 8.6 × 10^4^ cells/mL was immediately encapsulated into droplets and inoculated into 20 mL of LB medium for preparation of cell-free supernatant. *B*. *subtilis* from a glycerol stock was inoculated into 2 mL of LB medium and incubated with shaking at 30°C for 16 hours. The pre-cultured *B*. *subtilis* was sonicated for 1 minute at a power volume of 4 using an ultrasonic probe (Tomy Seiko Co., Ltd, Tokyo, Japan) on ice to disperse the culture into single cells. The concentration of *B*. *subtilis* was estimated using the number of colony-forming units (CFU) on an LB plate after overnight incubation. The pre-cultured *B*. *subtilis* was washed and resuspended in fresh LB medium. A dilution equivalent to 7.3 × 10^4^ CFU/mL was immediately encapsulated into droplets and inoculated into 20 mL of LB medium for preparation of cell-free supernatant. *S*. *aureofaciens* from a glycerol stock was inoculated into 2 mL of LB medium and incubated with shaking at 28°C for 3 days. The pre-cultured *S*. *aureofaciens* cells were sonicated for 2 minutes at a power volume of 4 using an ultrasonic probe (Tomy Seiko Co., Ltd) on ice to disperse the culture into single cells. The concentration of *S*. *aureofaciens* was determined as the CFU on an LB plate after overnight incubation. The pre-cultured *S*. *aureofaciens* was washed and resuspended in fresh LB medium. A dilution equivalent to 8.3 × 10^4^ CFU/mL was immediately encapsulated into droplets and inoculated into 20 mL of LB medium for preparation of cell-free supernatant. *B*. *japonicum* from a glycerol stock was inoculated into 2 mL of NBRC805 medium (1 g/L BactoYeast extract, 5 g/L mannitol, 0.7 g/L dipotassium hydrogen phosphate, 0.1 g/L potassium dihydrogen phosphate, and 1 g/L magnesium sulfate heptahydrate) and incubated with shaking at 28°C for 3 days in the dark. The concentration of *B*. *japonicum* was determined as the CFU on an NBRC805 plate after 6 days of incubation. The pre-cultured *B*. *japonicum* was washed and resuspended in fresh NBRC805 medium. A dilution equivalent to 3.8 × 10^4^ CFU/mL was immediately encapsulated into droplets and inoculated into 20 mL of NBRC805 medium for preparation of cell-free supernatant.

Soil samples were collected from the National Institute of Advanced Industrial Science and Technology in Japan (36° 06´ N, 140° 13´ E) in February 2018 and in January 2019. Five hundred milligrams of soil was directly used to extract genomic DNA (S-D0). About 5 g of soil was suspended in 30 mL of phosphate-buffered saline (PBS: 8 g/L NaCl, 0.2 g/L KCl, 3.6 g/L Na_2_HPO_4_ · 12H_2_O, 0.2 g/L KH_2_PO_4_; pH 7.4) and sonicated for 3 minutes at a power volume of 4 using an ultrasonic probe (Tomy Seiko Co., Ltd) on ice. The sonicated sample was centrifuged twice at 815 ×*g* for 5 minutes to remove soil particles. One milliliter of the supernatant was transferred to a new tube and centrifuged at 5,800 ×*g* at 4°C for 5 minutes in order to pellet the cells. The pellet was washed with PBS twice and re-suspended in 337 mL fresh LB medium in February 2018, which was immediately used as the inoculum for droplet culture and bulk culture, hereafter referred to SS-D0, or in 4.2 mL fresh LB medium in January 2019, which was used as the inoculum for droplet culture for imaging. One milliliter of SS-D0 was preserved at −20°C before DNA extraction.

### Preparation of cell-free medium

*E*. *coli* were grown with aeration at 37°C in 20 mL of LB medium. After 1 day of cultivation, 1 mL of culture medium was collected. *B*. *subtilis* were grown with aeration at 30°C in 20 mL of LB medium. After 1 day of cultivation, 1 mL of culture medium was collected. *S*. *aureofaciens* were grown with aeration at 28°C in 20 mL of LB medium. After 2 and 4 days of cultivation, 1 mL of culture medium was collected. *B*. *japonicum* were grown with aeration at 37°C in 20 mL of NBRC805 medium in the dark. After 6 days of cultivation, 1 mL of culture medium was collected. All of the collected culture samples were centrifuged to separate the supernatant, which was filtered through a 0.22-μm PVDF syringe filter (Merck Millipore, Darmstadt, Germany). The cell-free media were stored at −20°C until droplet generation.

### Bulk cultivation

Fifty microliters of SS-D0 was added to 50 mL sterilized LB medium and incubated with agitation at 25°C for 7 days. On days 1, 3, and 7, 1 mL culture medium was collected into a 1.5-mL tube and preserved at −20°C before DNA extraction.

### Droplet generation

FRET-based RNA probe (5'-Alexa488-UCUCGGUGCGUUG-BHQ1-3'; Japan Bio Services, Saitama, Japan) in the amount of 20 μL at 200 nM concentration mixed with each bacterial dilution, 20 μL of 200 nM FRET-based RNA probe mixed with 5 ng/μL RNase A (Merck Millipore), 20 μL of 200 nM FRET-based RNA probe, or 20 μL of 200 nM FRET-based RNA probe mixed with each cell-free medium was dispensed into a sample well of a DG8 cartridge (Bio-Rad Laboratories, Inc., Hercules, CA, USA). Then, 70 μL of 1% Pico-surf1 in Novec7500 (Dolomite, Royston, UK) was then loaded into each oil well of the DG8 cartridge, and the cartridge was covered with a DG8 gasket (Bio-Rad Laboratories, Inc.) and loaded into a QX100 droplet generator (Bio-Rad Laboratories, Inc.). Pico-surf1 dissolved in continuous oil, Novec7500, is a polyfluorinated surfactant, which stabilizes micro-droplets under a wide range of temperatures and biological conditions. The droplets in the emulsion wells of the DG8 cartridge were transferred into 1.5-mL tubes.

### Droplet cultivation

Droplets containing *E*. *coli* were statically incubated in 1.5-mL tubes at 37°C for 1 day. Droplets containing *B*. *subtilis* were statically incubated in 1.5-mL tubes at 30°C for 1 day. Droplets containing *S*. *aureofaciens* were statically incubated in 1.5-mL tubes at 30°C for 2 days. Droplets containing *B*. *japonicum* were statically incubated in 1.5-mL tubes at 28°C for 6 days. Droplets containing bacteria collected from soil were statically incubated in 1.5-mL tubes at 25°C for 7 days and sorted on days 1, 3, and 7.

### Droplet imaging

The droplets were placed into a μ-Slide VI flat uncoated microscopy chamber (IBIDI, Martinsried, Germany) prefilled with 1% Pico-surf1 in Novec7500. Optical and fluorescence images were obtained using an Axio Imager 2 (Carl Zeiss, Jena, Germany) equipped with a DP72 camera (Olympus, Tokyo, Japan) operated by DP2-BSW software (Olympus).

### Droplet sorting

Fluorescent measurement and droplet sorting were performed using On-chip Sort (On-chip Biotechnologies, Tokyo, Japan). On-chip Sort is a microfluidic-based sorting system equipped with a disposable chip, which enables the use of fluorocarbon oil as a sheath liquid. On-chip Sort with a blue laser with an excitation of 30 mW at 488 nm can detect the forward scatter and green fluorescence (532–554 nm) from individual droplets. Eight milliliters and 1.5 mL of sheath liquid oil, obtained by diluting 5% Pico-surf1 in Novec7500 (Dolomite) with 50 volumes of Droplet Reader oil (Bio-Rad Laboratories, Inc.), were added to the sheath reservoir and sorting reservoir, respectively, in a 150-μm wide disposable sorting chip (On-chip Biotechnologies). One hundred microliters of 1% Pico-surf1 in Novec7500 (Dolomite) was added to the collection reservoir of the chip. The sample pressure and sheath pressure were fixed at 0.09 psi and 0.07 psi, respectively, resulting in a sample flow rate of 30–120 events per second. The threshold line on the fluorescence histogram was arbitrarily defined to collect a highly fluorescent droplet population. The droplets within the gated histogram area were collected into the collection reservoir, and the droplets within the un-gated histogram area flowed into the waste reservoir in the chip. After sorting the droplets containing the model bacteria, the droplets collected in the collection reservoir were observed using dark-field and fluorescence microscopy or were used to obtain a CFU count, while those in the waste reservoir were discarded. After sorting the droplets containing the soil bacteria, the droplets collected in the collection reservoir were transferred into 1.5-mL tubes and stored at −20°C. The droplets in the waste reservoir were transferred into 1.5-mL tubes, iteratively cultivated and sorted on days 1 and 3, and stored at −20°C on day 7. Analysis of the data output from On-chip Sort was performed with FlowJo software v10.4.2 (FlowJo, Ashland, OR, USA).

### DNA extraction

The droplets were broken by freezing and thawing to extract genomic DNA from the cells present in the droplet samples. The aqueous phase containing bacterial cells was used for DNA extraction. SS-D0 and the frozen bulk culture samples (in bulk cultivation for 1, 3, and 7 days) were thawed, and the pellets were collected by centrifugation at 5,800 ×*g* for 3 minutes, 8,000 ×*g* for 6 minutes, and 12,000 ×*g* for 3 minutes. The DNA was extracted from the soil sample, SS-D0, the aqueous phases of droplets, and bulk cultures using the FastDNA SPIN Kit for Soil (MP Biomedicals, Santa Ana, CA, USA) according to the manufacturer’s instructions.

### Construction of 16S rRNA gene library and sequencing

The V4 region of the 16S rRNA gene was amplified with specific primers 515F/806R [26]. The forward primer, which was adapted for the Illumina MiSeq platform by the addition of nine extra bases, included the Illumina flowcell adaptor, and the reverse primer included the Illumina flowcell adaptor and 12-bp barcodes unique to each sample. Each reaction contained 1 μL of 10× diluted or undiluted template DNA, 1× Ex Taq buffer (Takara Bio Inc., Shiga, Japan), 0.2 mM of each dNTP, 0.5 μM of the 515F and 806R primers, and 0.5 U of Ex Taq polymerase (Takara Bio Inc.) in a total volume of 20 μL. PCR was conducted under the following conditions: 40 cycles at 98°C for 10 seconds, at 50°C for 30 seconds, and at 72°C for 1 minute. Three separate PCR products for each sample were mixed to minimize the effect of potential early-round PCR errors [27]. PCR products were purified using an Agencourt AMPure XP (Beckman Coulter Inc., Brea, CA, USA) and quantified using an Agilent Bioanalyzer D1000 (Santa Clara, CA, USA). The PCR amplicon libraries were pooled and sequenced using the 2 × 250 bp paired-end MiSeq platform with the v2 reagent kit (Illumina Inc., San Diego, CA, USA).

### Post-sequencing analysis

MiSeq paired-end sequencing generated three files per sample: a forward read file, a reverse read file, and an index read file. All forward and reverse reads were trimmed to include only 200 bases or less using the fastx_trimmer script in the FASTX-Toolkit (http://hannonlab.cshl.edu/fastx_toolkit/). The trimmed paired-end reads were assembled with a minimum overlap of 20 bp by PANDAseq [28]. All assembled sequences were processed with Quantitative Insights Into Microbial Ecology (MacQIIME v1.9.1) [29]. The assembled sequences were demultiplexed by sample using the split_libraries_fastq.py script, and chimeric sequences were identified using the identify_chimeric_seqs.py script with the -m usearch61 option. The chimeric sequences were eliminated, and the remaining sequences were clustered into operational taxonomic units (OTUs) at a sequence identity cut-off value of 97% using the filter_fasta.py and pick_otus.py scripts, respectively. The most abundant reads from each OTU cluster were selected as representative sequences using the pick_rep_set.py script with the -m most_abundant option; these were used for taxonomical identification. Taxonomy was assigned using the Greengenes 13_8 database and UCLUST algorithm using the assign_taxonomy.py script with default parameters. OTUs were counted using make_otu_table.py and summarized using summarize_taxa.py. These sequence data have been submitted to the DDBJ database under accession number DRA007503 and DRA007504.

### Viability of bacteria cultivated in droplets

After sorting 1,000 droplets containing model bacteria, the droplets were collected into 1.5-mL tubes. Then 99 μL of LB medium and surfactant-free Novec7500 (3M, St Paul, MN, USA) was added to the 1,000 sorted droplets containing *E*. *coli*, *B*. *subtilis*, or *S*. *aureofaciens*. An additional 99 μL of NBRC805 medium and surfactant-free Novec7500 (3M) was added to the 1,000 sorted droplets containing *B*. *japonicum*. Until separation of oil and aqueous phases, the droplets were repetitively washed with surfactant-free Novec7500. The aqueous phase containing *E*. *coli*, *B*. *subtilis*, or *S*. *aureofaciens* cells was serially diluted and plated on duplicate LB plates for CFU determination. The aqueous phase containing *B*. *japonicum* cells was serially diluted and plated on duplicate NBRC805 plates for CFU determination.

## Results

### The FNAP-sort method

**Fig 1:**
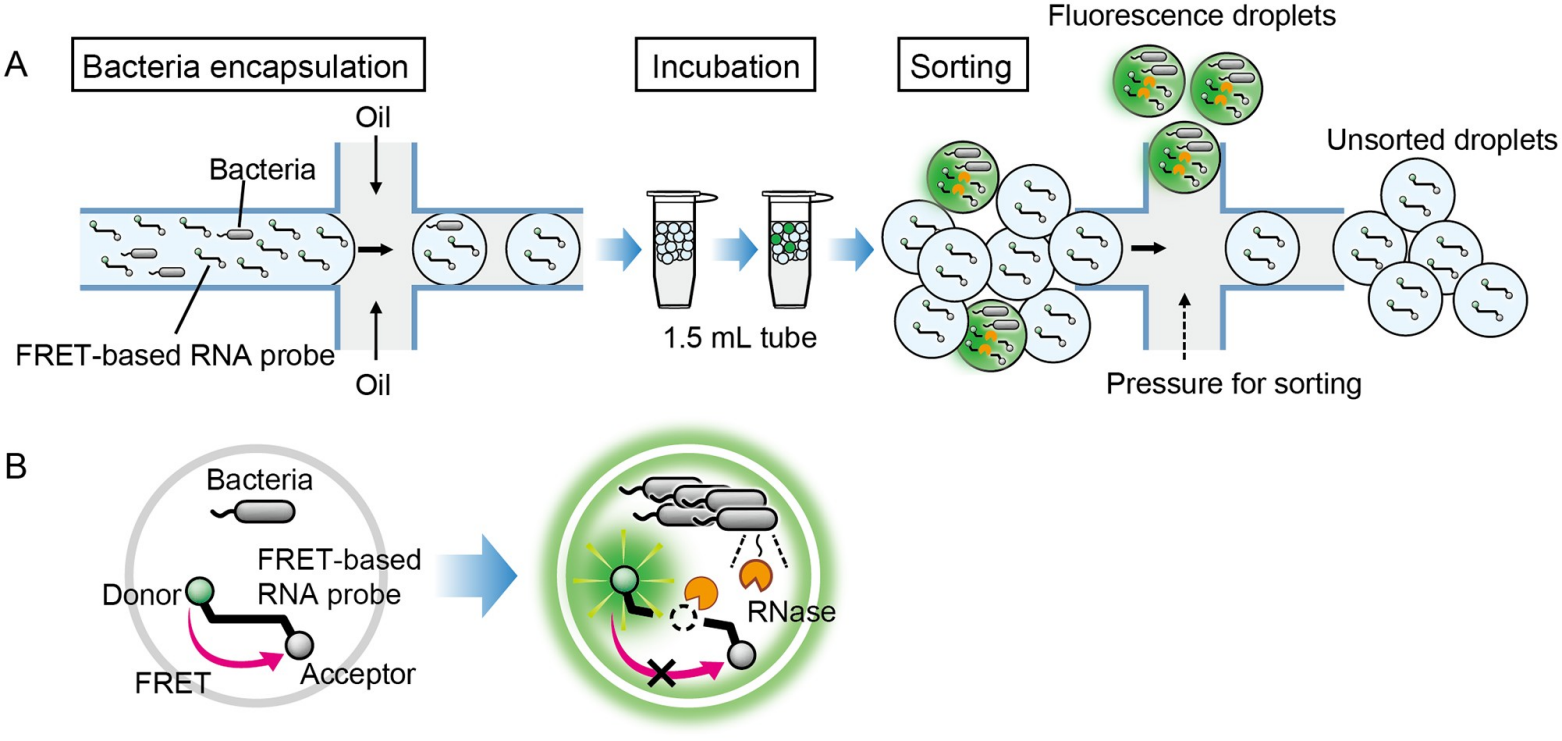
Schematic images of the FNAP-sort method. (A) The workflow of FNAP-sort consists of three steps: 1) Encapsulation of bacterial cell(s) mixed with FRET-based RNA probes into 1-nL droplets, 2) Incubation of the droplets in a 1.5-mL tube to allow for cell growth and the probe cleavage reaction, and 3) Sorting of highly fluorescent droplets containing growing bacteria. (B) A FRET-based RNA probe generates strong fluorescence upon RNA cleavage by RNases secreted from growing bacteria in a droplet.

We developed a novel droplet sorting system, FNAP-sort, consisting of three steps (Fig 1A). In the first step, a FRET-based RNA probe is encapsulated with a single bacterial cell or bacterial cells suspended in culture medium into a 1-nL droplet. In the second step, the droplets are statically incubated in a 1.5-mL tube to allow encapsulated bacterial cells to grow. The droplets with growing bacteria emit fluorescence by the cleavage of the FRET-based RNA probe. In the third step, the droplets are sorted according to fluorescence intensity using On-chip Sort, which is a bench-top sorting device equipped with a disposable microfluidic chip.

The FRET-based RNA probe used here is a short RNA oligoribonucleotide labeled with Alexa488 at the 5' end and with Black Hole Quencher1 (BHQ1) at the 3' end (Fig 1B). Since the Alexa488 and BHQ1 are in close proximity, an intact probe is normally quenched. When the FRET-based RNA probe is cleaved by an RNase derived from growing bacteria, the two dyes are spatially separated, resulting in an increase in fluorescence. Therefore, using FNAP-sort, droplets containing growing bacterial cells can be selectively sorted on the basis of droplet fluorescence intensity.

**Fig 2:**
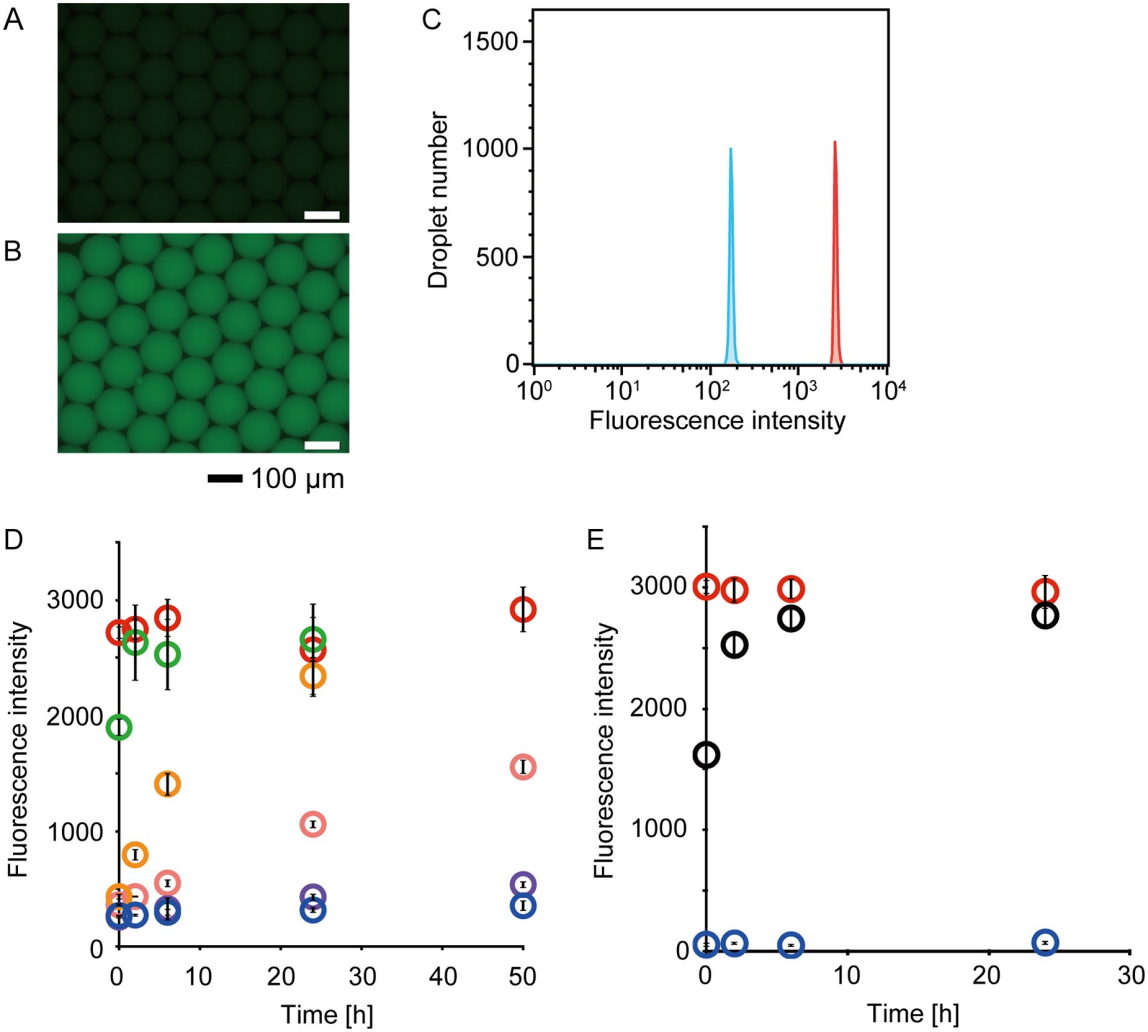
RNase activity measurements using FRET-based RNA probe. The droplets containing FRET-based RNA probe (A) with and (B) without commercial RNase A were observed using fluorescence microscopy. (C) Histograms obtained from On-chip Sort analysis describe the distributions of the fluorescence intensities of the droplets containing FRET-based RNA probe with (blue line) and without (red line) commercial RNase A. (D) Averages of fluorescence intensity of approximately 1,000 droplets analyzed by On-chip Sort. Blue circles, droplets containing LB medium. Red circles, droplets containing LB medium, FRET-based RNA probe, and RNase A. Orange circles, droplets containing crude supernatant of *E*. *coli* cultivated for 1 day and FRET-based RNA probe. Green circles, droplets containing crude supernatant of *B*. *subtilis* cultivated for 1 day and FRET-based RNA probe. Pink circles, droplets containing crude supernatant of *S*. *aureofaciens* cultivated for 2 days and FRET-based RNA probe. Purple circles, droplets containing crude supernatant of *S*. *aureofaciens* cultivated for 4 days and FRET-based RNA probe. (E) Averages of fluorescence intensity of approximately 1,000 droplets analyzed by On-chip Sort. Blue circles, droplets containing NBRC805 medium. Red circles, droplets containing NBRC805 medium, FRET-based RNA probe, and RNase A. Black circles, droplets containing crude supernatant of *B*. *japonicum* cultivated for 6 days and FRET-based RNA probe.

To demonstrate the detection of the droplets via fluorescence emission induced by RNA cleavage, fluorescent droplets, which contain the FRET-based RNA probe and commercially available RNase A in LB medium, and quenching droplets, which contain only the FRET-based RNA probe in LB medium, were prepared. Microscopic observation in fluorescence mode clearly distinguished the fluorescent droplets from the quenching droplets (Fig 2A and 2B). The fluorescence intensity of individual droplets was measured with On-chip Sort. As a result, fluorescence intensities of the fluorescent droplets and quenching droplets exhibited single-peaked distributions (Fig 2C). The mean of the fluorescence intensities of the fluorescent droplets was 14.5-fold higher than that of the quenching droplets. Thus, the fluorescent droplets could be discriminated from the quenching droplets on the basis of their fluorescence intensity. In order to corroborate that the bacteria secrete RNase and that the secreted RNase induces fluorescence emission, crude supernatants of the four model bacteria were incubated with FRET-based RNA probe in 1-nL droplets. The fluorescence intensities of the cell-free media of *E*. *coli*, *B*. *subtilis*, or *B*. *japonicum* incubated with the FRET-based RNA probe were almost equivalent to that of LB medium mixed with commercial RNase and the FRET-based RNA probe (Fig 2D and 2E). The fluorescence intensity of the cell-free medium of *S*. *aureofaciens* cultivated for 2 days and reacted with the FRET-based RNA probe for 48 hours (537 ± 29.9) was slightly higher than that of LB reacted with FRET-based RNA probe for 48 hours (354 ± 45.1). Additionally, the fluorescence intensity of the cell-free medium of *S*. *aureofaciens* cultivated for 4 days and reacted with FRET-based RNA probe for 48 hours (1555 ± 60.7) was distinctly higher than those of both LB with FRET-based RNA probe and the cell-free medium of *S*. *aureofaciens* cultivated for 2 days and reacted with FRET-based RNA probe. However, the fluorescence intensity value of the cell-free medium of *S*. *aureofaciens* did not reach that of LB with commercial RNase and FRET-based RNA probe. Accordingly, we confirmed that the fluorescence intensities of droplets containing the cell-free medium of each culture, including secreted RNase, and the FRET-based RNA probe increased over time.

### Detection of model bacteria in droplets using FRET-based RNA probe

**Fig 3:**
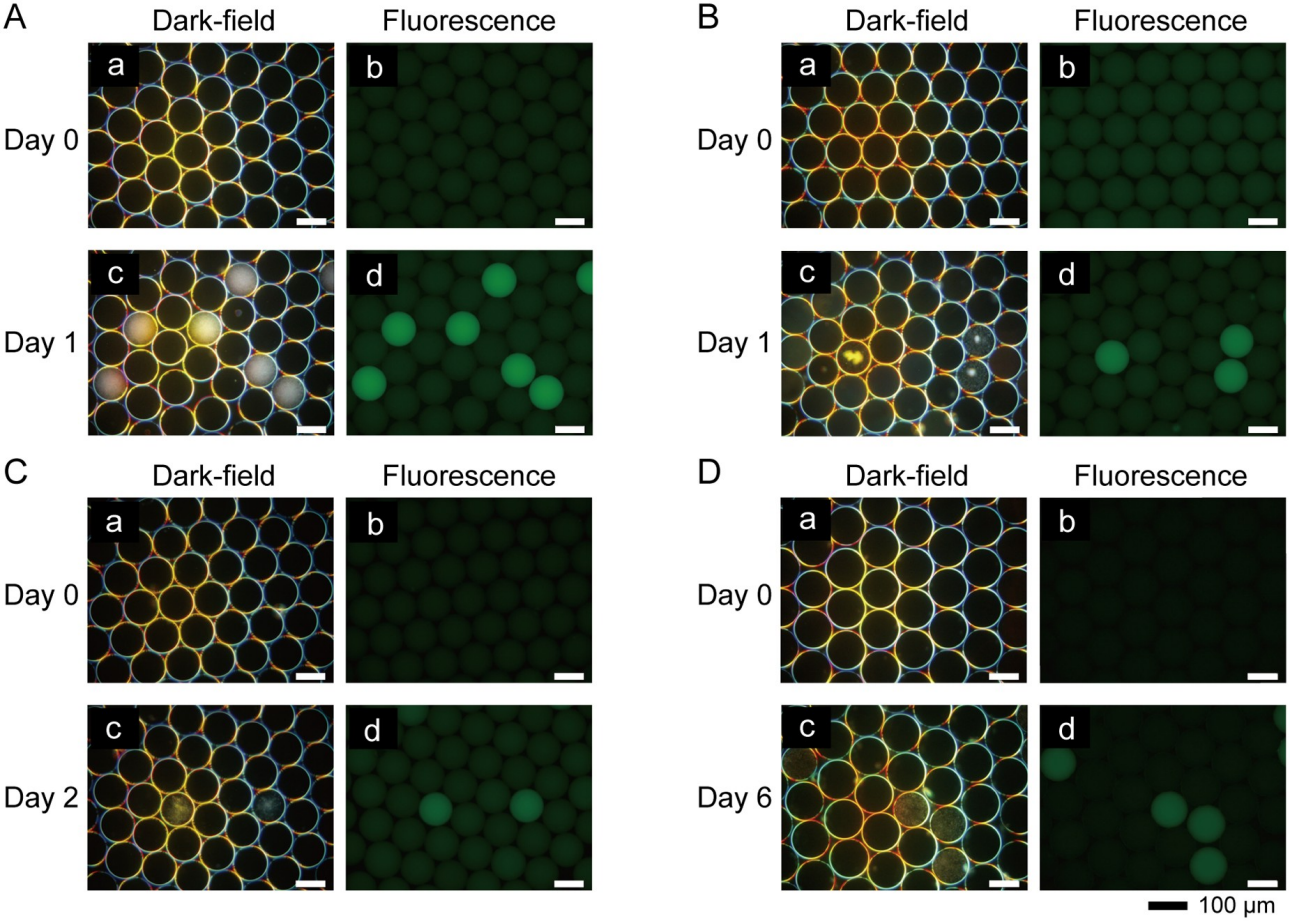
Representative micrographs of droplets with model bacteria and FRET-based RNA probes. (a) Dark-field and (b) fluorescence micrographs showing droplets immediately after bacterial encapsulation. (c) Dark-field and (d) fluorescence micrographs showing droplets containing bacteria after incubation. (A) *E*. *coli* before and after 1 day of incubation. (B) *B*. *subtilis* before and after 1 day of incubation. (C) *S*. *aureofaciens* before and after 2 days of incubation. (D) *B*. *japonicum* before and after 6 days of incubation. Dark-field and fluorescence images from the same day show the same microscopic fields.

To demonstrate the utility of FNAP-sort, *E*. *coli* was used as a model bacterium. To encapsulate one or more cells in 1-nL droplets, pre-cultured *E*. *coli* cells were diluted with fresh LB medium to 8.6 × 10^4^ cells/mL. The diluted *E*. *coli* suspension was encapsulated into 1-nL droplets with the 200 nM FRET-based RNA probe. Fluorescence microscopic observation of the droplets revealed that all droplets emitted weak fluorescence immediately after droplet generation (Fig 3Ab). The droplets were statically incubated at 37°C for 1 day to allow for cell growth, and *E*. *coli* growth was observed in some of the droplets using dark-field microscopy (Fig 3Ac). Furthermore, after 1 day of incubation, some of the brighter droplets were observed using fluorescence microscopy (Fig 3Ad). Dark-field and fluorescence micrographs revealed that the brighter droplets were those that contained growing *E*. *coli*. To test other model bacteria, *B*. *subtilis*, *S*. *aureofaciens*, and *B*. *japonicum* were also encapsulated into the droplets with the FRET-based RNA probe, and droplets were observed with fluorescence microscopy immediately after droplet generation (Fig 3Bb, 3Cb and 3Db). After static incubation for 1, 2, or 6 days, all of the brighter droplets contained growing bacteria (Fig 3Bc, 3Bd, 3Cc, 3Cd, 3Dc and 3Dd). While *E*. *coli* and *B*. *japonicum* grew entirely in droplets, *B*. *subtilis* and *S*. *aureofaciens* aggregated in droplets (Fig 3Ac, 3Bc, 3Cc and 3Dc). All droplets containing growing bacterial cells were successfully detected via fluorescence.

### Sorting of droplets containing model bacteria using FNAP-sort

**Fig 4:**
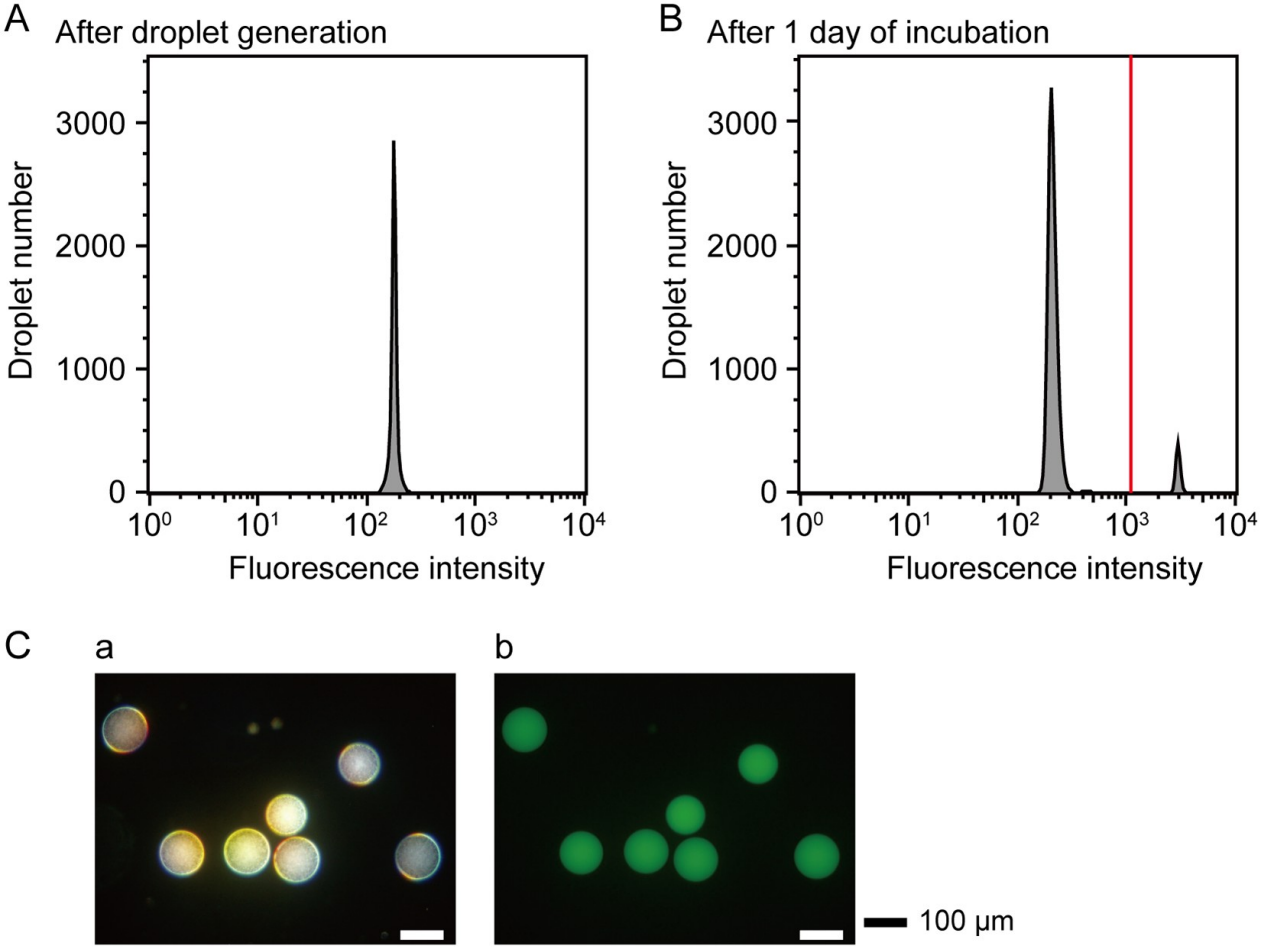
Sorting of droplets with growing *E*. *coli*. Histograms describe the distributions of droplet fluorescence intensities (A) immediately after droplet generation and (B) after 1 day of incubation. The red line represents the sorting threshold. (C) (a) Dark-field and (b) fluorescence micrographs show the sorted droplets with fluorescence intensities above the red threshold line.

We next tested the feasibility of FNAP-sort for the sorting of droplets containing growing bacterial cells via fluorescence using *E*. *coli*. The fluorescence intensities of the droplets immediately after *E*. *coli* encapsulation and after 1 day of incubation for cell growth were measured using On-chip Sort, in which oil can be used as a sheath liquid when droplet sorting. Immediately after droplet generation, histogram analysis using FlowJo software resulted in a distinct unimodal distribution (Fig 4A). The generated droplets were statically incubated in 1.5-mL tubes at 37°C for 1 day, and then droplet fluorescence intensity was measured using On-chip Sort. Fluorescent measurement resulted in a bimodal distribution, consisting of the populations of droplets with and without growing *E*. *coli* (Fig 4B). The population of 1,500 brightly fluorescent droplets was selectively sorted with On-chip Sort from a total of 22,667 droplets (S1 Table). Most of the sorted droplets emitted strong fluorescence and contained growing *E*. *coli* (Fig 4C). *B*. *subtilis*, *S*. *aureofaciens*, and *B*. *japonicum* were also encapsulated into 1-nL droplets and incubated to allow for cell growth under each culture condition. Immediately after droplet generation, the fluorescence intensities of the droplets containing *B*. *subtilis*, *S*. *aureofaciens*, and *B*. *japonicum* showed single peaks in each histogram (S1A, S1E and S1I Fig). After 1, 2, or 6 days of incubation, respectively, the histograms acquired from On-chip Sort analysis also exhibited bimodal distributions (S1B, S1F and S1J Fig). Populations with fluorescence intensities above the threshold were sorted using On-chip Sort, and the sorted droplets were observed (S1 Table). All sorted droplets had strong fluorescence and contained bacteria (S1C, S1D, S1G, S1H, S1K and S1L Fig). After droplet sorting, to verify that the four model bacteria are capable of regrowth after growing in droplets, the CFUs per droplet were determined. A total of 12,400, 590, and 2,500 CFUs/droplet were successfully recovered from the droplets containing *E*. *coli*, *B*. *subtilis*, and *B*. *japonicum*, respectively (S2 Table). Unexpectedly, we obtained a CFU value of *S*. *aureofaciens* of less than 1 CFU/droplet, indicating that the proliferation activity of *S*. *aureofaciens* recovered from droplets after 2 days of incubation was weak. Consequently, these results indicate that cells capable of growing after droplet cultivation could be recovered.

### Sorting of droplets containing soil bacteria using FNAP-sort

**Fig 5:**
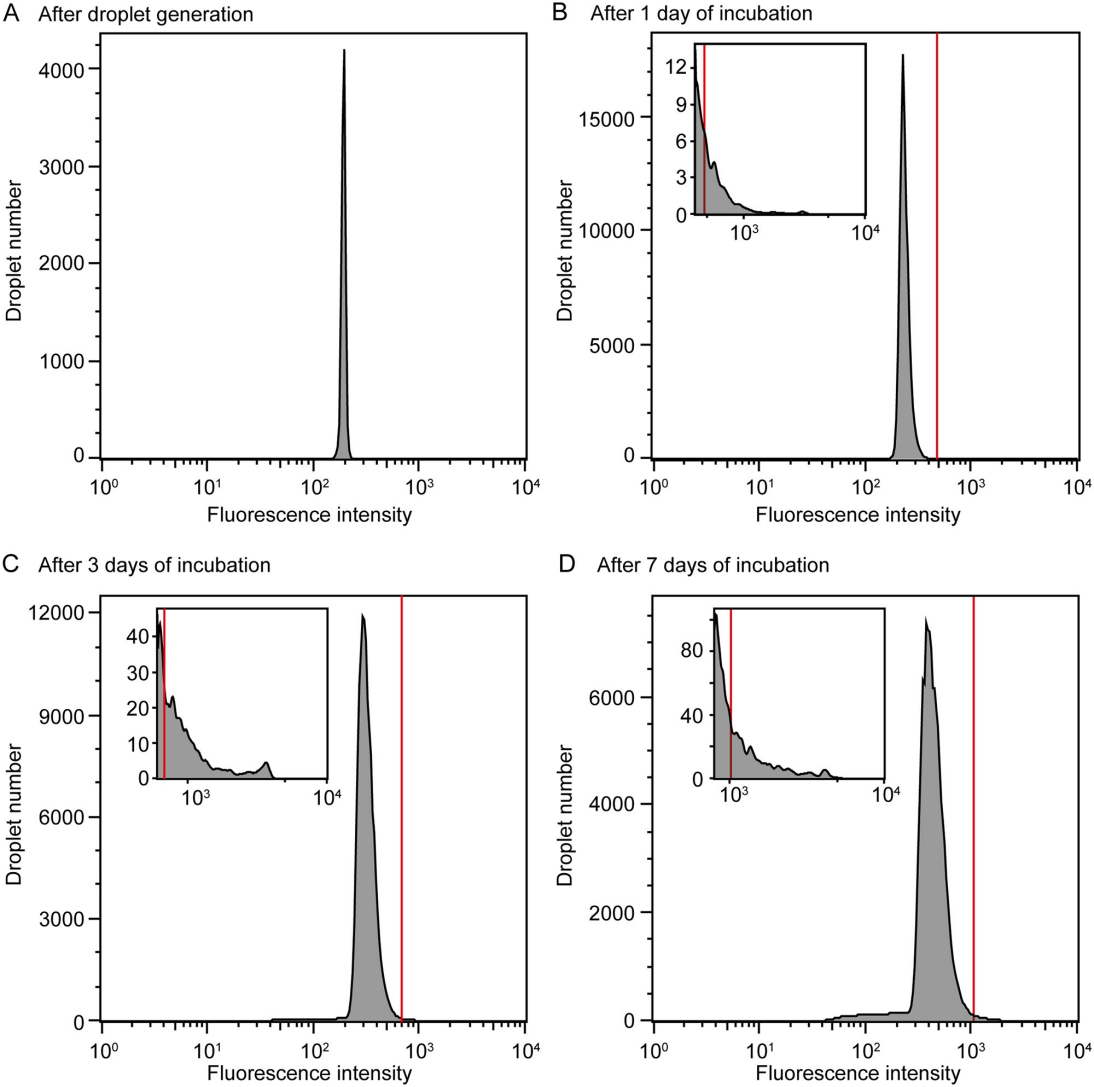
Sorting of droplets containing soil bacteria. Histograms show the distributions of droplet fluorescence intensities after (A) 0, (B) 1, (C) 3, and (D) 7 days of cultivation. The red lines represent the sorting threshold. Each inset shows a histogram of fluorescence intensity above (B) 400, (C) 600, and (D) 800.

We next applied FNAP-sort to the sorting of soil bacteria collected in February 2018. On day 0, approximately 530,000 droplets were generated using the SS-D0 bacterial solution, which was prepared from soil by sonication, centrifugation, and dilution. Droplet fluorescence measurements taken with On-chip Sort resulted in a uniform histogram immediately after droplet generation (Fig 5A and S2A Fig). On day 1, the fluorescence intensities of all 453,923 of the incubated droplets were measured using the On-chip Sort, and 841 droplets that were above the threshold (SD-D1) were sorted (Fig 5B, S2B Fig and Table 1). On day 3, 3,350 droplets above the threshold (SD-D3) were sorted from the 409,160 re-incubated droplets that were unsorted on day1 (Fig 5C, S2C Fig and Table 1), and on day 7, 4,775 droplets above the threshold (SD-D7) were sorted from the 378,596 re-incubated droplets that were unsorted on day 3 (Fig 5D, S2D Fig and Table 1). The sorted droplets from days 1, 3, and 7 and the unsorted droplets on day 7 (UD-D7) were preserved at −20°C until DNA extraction. To compare the bacterial composition obtained using a bulk culture system with that obtained using the droplet culture system, we also extracted DNA from 1 mL of culture sample collected from bulk culture on days 1 (BC-D1), 3 (BC-D3), and 7 (BC-D7). In another experiment in January 2019, droplets containing soil bacteria and the FRET-based RNA probe were generated and statically incubated for cell growth. After 7 days of incubation, a few droplets in which soil bacteria grew emitted strong fluorescence (S3A and S3B Fig). The droplets with high fluorescence intensity were sorted (S3C Fig). Micrographs subsequently showed that most of the sorted droplets contained growing cells, supporting the theory that the sorting process enriched the droplets in those containing growing cells (S3D and S3E Fig).

**Table 1:** Analysis of the FNAP-sort applied to the bacteria from soil.

	Day 0	Day 1	Day 3	Day 7
Total droplet number	530,462	453,923	409,160	378,956
Sorted droplet number		841	3,350	4,775
Percentage of sorted droplets (%)		0.19	0.82	1.26

Bacterial community structures were analyzed on the basis of 16S rRNA gene sequences using the MiSeq platform. As shown in S3 Table, the OTUs in each sample were generated using 176,781–351,016 effective reads. The highest number of OTUs was obtained from S-D0 (47,728 OTUs), while the lowest number of OTUs was found in BC-D7 (6,440 OTUs). The number of OTUs in the unsorted droplets on day 7 (13,145 OTUs) was much higher than that in the sorted droplets on days 1 (9,441 OTUs), 3 (8,094 OTUs), and 7 (7,730 OTUs). In contrast, the number of OTUs in bulk culture on days 3 (7,654 OTUs) and 7 (6,440 OTUs) dramatically decreased from that on day 1 (13,101 OTUs).

**Fig 6:**
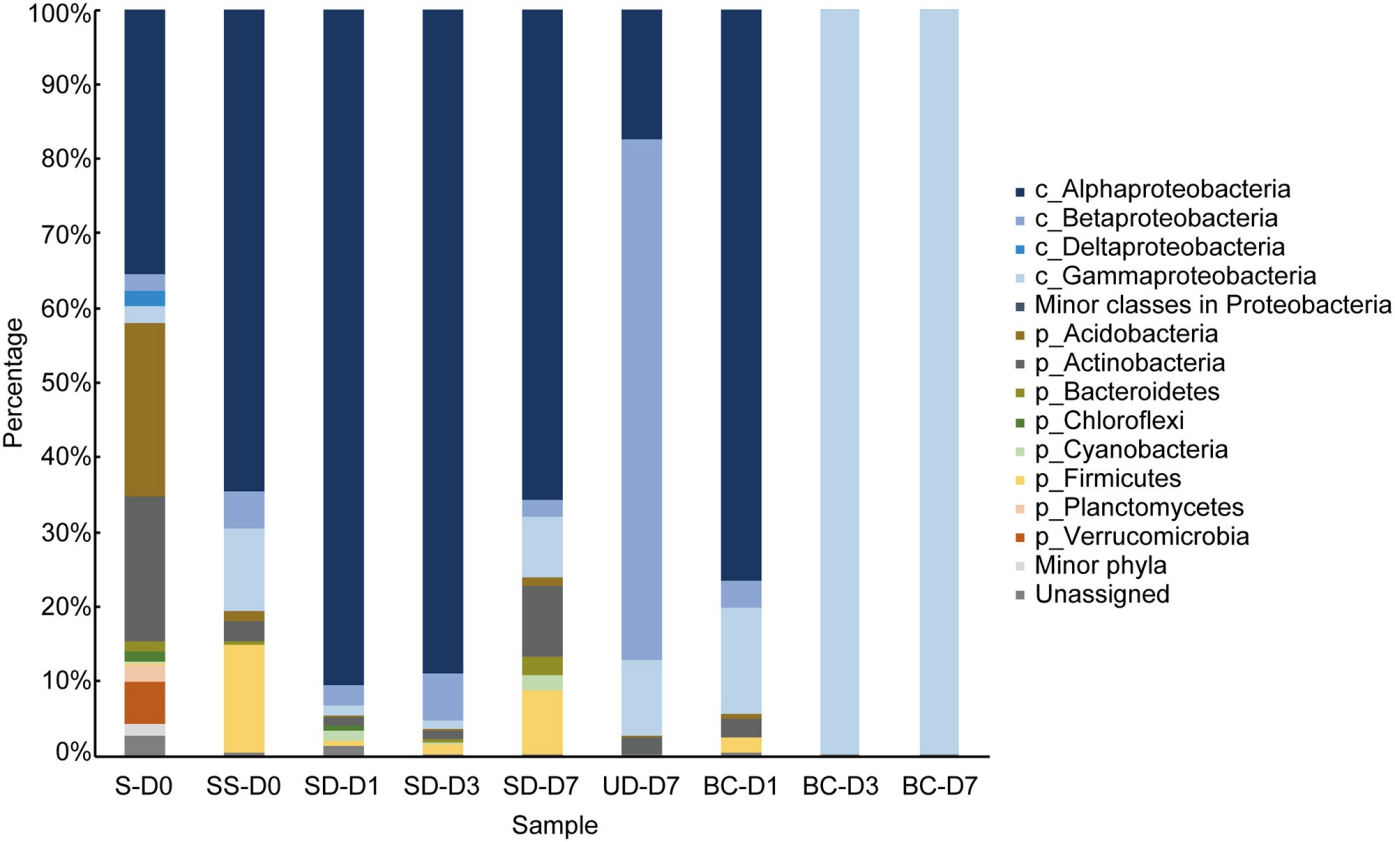
Abundances and distributions of major phyla and major classes within Proteobacteria. Taxa with a relative abundance above 1% in at least one sample are shown. Minor phyla and minor classes within Proteobacteria refer to taxa with abundances below 1% in all samples.

As shown in Fig 6, Proteobacteria was the most abundant phylum in all samples. In S-D0, Proteobacteria accounted for 42% of all OTUs, with the second and third most abundant phyla being Acidobacteria (23%) and Actionobacteira (23%), respectively. The percentages of Proteobacteria in other samples were 76–99%. In the SS-D0, Acidobacteria and Actinobacteria exhibited low abundances, while Firmicutes was detected at a rate of 14%. In comparison to the SD-D1 and SD-D3 samples, in which Proteobacteria accounted for over 90%, the percentage of Proteobacteria in SD-D7 decreased to 76%, while those of Firmicutes (9%) and Actinobacteria (10%) increased. While the bacterial composition of BC-D1 was similar to that of SS-D0, the percentage of Proteobacteria reached more than 99% in BC-D3 and BC-D7.

Within the Proteobacteria, in S-D0, Alphaproteobacteria was the major class, with Betaproteobacteria, Deltaproteobacteira, and Gammaproteobacteria accounting for similar proportions (approximately 2%). Except for in S-D0, Gammaproteobacteria was only minimally detected (less than 0.01%). In SS-D0, SD-D1, SD-D3, and SD-D7, the relative abundance of the predominant class, Alphaproteobacteria, in the sorted droplets gradually decreased, with values of 90% on day 1, 89% on day 3, and 66% on day 7. Intriguingly, Betaproteobacteria, which was detected at a rate of less than 7% in all samples except for UD-D7, accounted for approximately 70% in UD-D7. Whereas the BC-D1 sample contained Alphaproteobcteria (77%), Betaproteobacteria (4%), and Gammmaproteobacteria (14%), Gammaproteobacteria was dominant in BC-D3 and BC-D7, while other classes of Proteobacteria accounted for less than 1% in BC-D3 and BC-D7.

## Discussion

In the present study, we developed a method for the sorting of droplets containing growing bacterial cells that involved using a FRET-based RNA probe, and we named this method FNAP-sort. In FNAP-sort, RNases secreted from growing bacteria cleave the FRET-based RNA probes in water-in-oil droplets, resulting in strong fluorescence emission. The increase in the fluorescence intensity of droplets should be caused by the degradation kinetics of the FRET-based RNA probe. We presume that the probe degradation kinetics depend on the amounts and activities of RNases in the droplets. The amounts and activities of RNases are related to bacterial growth rate and growth phase, RNase secretory capacity, and RNases type, for which bacterial species vary. *E*. *coli* has several types of RNases, including RNase I, RNase III, and RNase E [30, 31]. RNase I is a nonspecific endoribonuclease and resides in the periplasmic space in vivo [32]. Therefore, RNase I should function outside of the cell, similar to alkaline phosphatase, which resides in the periplasmic space of gram-negative bacteria [32, 33]; RNase I should therefore cleave the FRET-based RNA probe in the droplet, resulting in fluorescence emission. In addition, RNase Bsn [34] and RNase Sa [35], which are extracellular ribonucleases, were discovered in *B*. *subtilis* and *S*. *aureofaciens*, respectively. Consequently, the amounts and activities of RNase secreted would be expected to differ from species to species. Indeed, the supernatant of *B*. *subtilis* after 1 day of cultivation and that of *S*. *aureofaciens* after 2 days of cultivation showed different probe degradation activities (Fig 2D). Therefore, the fluorescence intensity of each droplet may provide interesting information.

Our results showed that highly and weakly fluorescent droplets could be distinguished for at least 6 days. We hypothesized that the FRET-based RNA probe used in FNAP-sort would be hydrophilic and thus less likely to transfer from an aqueous droplet to the oil phase and adjacent droplets. Importantly, droplets were also stabilized against coalescence for at least 6 days. The FRET-based RNA probe and droplets stabilized by the polyfluorinated surfactant Pico-surf1 are therefore suitable for the cultivation of slow-growing microbes that require longer incubation times. While the FRET-based RNA probe is stably maintained in the droplets, the fluorescence intensities of empty droplets after 6 days of incubation increased slightly compared to the initial fluorescence intensities. The reason for this slight increase in the fluorescence intensity of empty droplets may be explained by the sensitivity of the FRET-based RNA probe to contamination with RNase from the medium, such as from yeast extract. Since the resistance of the FRET-based RNA probe to degradation is necessary for the cultivation of slow-growing species needing long incubation times, the composition of the medium, which is susceptible to RNase contamination, should be improved. In this study, the droplets containing environmental bacteria in the top approximately 0.1–1% in fluorescence histograms were sorted (Fig 5B, 5C and 5D; S2B, S2C and S2D Fig). The fluorescence histograms of the four model bacteria exhibited apparent bimodal distributions. In contrast, those of environmental samples exhibited a single broad peak, making it difficult to determine the threshold value. Therefore, the determination of threshold values should be validated in the future.

In analyzing the bacterial compositions of our soil sample, the relative abundances of the three dominant phyla, Actinobacteria, Acidobacteria, and Proteobacteria, were similar to those reported by Janssen [36]. It is noteworthy that the number of OTUs in SS-D0 was significantly lower than that in S-D0 and that the bacterial composition of SS-D0 was markedly different from that of S-D0. SS-D0 was prepared by washing S-D0 with PBS, followed by sonication and centrifugation to collect the supernatant. These processes are therefore the causes of this reduction in bacterial diversity; sonication in particular may affect the collection efficiency, as soil bacteria may adhere strongly to soil particles. Since the bacterial diversities of cultures are dependent on that of the initial inoculate, the preparation of environmental samples for droplet culture leaves room for improvement.

It was surprising that the bacterial composition of SD-D7 was similar to that of SS-D0. This result suggested that the compartmentalized droplets maintained their initial bacterial composition after 7 days of incubation, while the bulk culture became enriched in Gammaproteobacteria after 3 days of incubation. The number of OTUs in UD-D7 was much higher than those in the sorted droplet samples and bulk culture samples. UD-D7 contained 373,821 unsorted droplets and therefore more droplets than were found in the sorted samples on days 1, 3, and 7, resulting in an increase in the OTU number. Most importantly, the bacterial composition of UD-D7 was significantly different from those of the sorted droplet samples and bulk culture samples. The bacteria detected from UD-D7 were unable to cleave the FRET-based RNA probe after 7 day of cultivation. It has been reported that long incubation times enhance the colony formation and cultivation of rarely isolated species [37]. This knowledge suggests that the longer the droplets are incubated, the more species can be sorted. The ability of microbes to secrete RNases is required for the success of FNAP-sort. Therefore, bacteria incapable of secreting RNases into the environment are unlikely to be detected based on the fluorescence caused by the cleavage of the FRET-based RNA probe. It was not determined whether the bacteria included in UD-D7 were alive, dead, or unable to grow in the droplets. Investigations into the bacteria in these unsorted droplets would be useful to demonstrate the potential for droplet cultivation.

The droplet culture system represents a potential technique for bacterial isolation. Although agar plates have been used for the isolation of microbes previously, streaked microbes on agar plates compete for nutrients and space. Compared to traditional agar-based cultivation, the cultivation spaces of droplet culture are separated by the oil phase; therefore, compartmentalization prevents hydrophilic molecules, such as amino acids, from being transferred between droplets [38]. In FNAP-sort, the bacterial number per droplet can be controlled by adjusting the bacterial concentration in the aqueous phase solution prepared during the encapsulation process. Single-cell encapsulation is effective for pure culture isolation, while co-culture is a useful strategy for isolating bacteria that require products from other bacteria to grow [39]. Although the necessary components of the products remain unclear, it has been reported that bacterial growth is stimulated by the addition of supernatants from other bacterial cultures [37, 40]. In the droplet culture system, bacteria collected from the natural environment can be randomly distributed in various combinations; thus, it may be possible to efficiently explore unknown symbiotic microorganisms in a high-throughput manner. In this study, we confirmed that cells capable of growing after droplet cultivation could be recovered. However, the CFU of *S*. *aureofaciens* cultivated in droplets was less than 1 per droplet, presumably due to the loss of and damage to cells during the recovery process. In the future, the method for recovering bacteria efficiently and gently from sorted droplets should be improved.

In this report, we described a novel method for selectively sorting droplets containing growing bacteria on the basis of their fluorescence emission due to the cleavage of a FRET-based RNA probe. The FRET-based RNA probe is cleaved by RNase, an essential enzyme produced by all organisms. Although there are few reports on extracellular ribonucleases from environmental microorganisms, our results suggest that detection of RNase activity is suitable for the sorting of droplets containing microorganisms. Furthermore, to the best of our knowledge, the method described herein is the first droplet culture system that enables iterative cultivation and sorting. Therefore, FNAP-sort has potential for the high-throughput cultivation and isolation of environmental samples in droplets.

## Supporting information

S1 Fig Sorting of droplets with growing *B*. *subtilis*, *S*. *aureofaciens*, and *B*. *japonicum*. (A–D) Droplets with *B*. *subtilis* were analyzed and sorted by On-chip Sort. Histograms describe the distributions of the fluorescence intensities of the droplets (A) immediately after droplet generation and (B) after 1 day of incubation. Microscopic images in (C) bright-field and (D) fluorescence mode show the sorted droplets with fluorescence intensities above the red threshold line in S1B Fig. (E–H) Droplets with *S*. *aureofaciens* were analyzed and sorted by On-chip Sort. Histograms describe the distributions of the fluorescence intensities of the droplets (E) immediately after droplet generation and (F) after 2 days of incubation. Microscopic images in (G) bright-field and (H) fluorescence mode show the sorted droplets with fluorescence intensities above the red threshold line in S1F Fig. (I–L) Droplets with *B*. *japonicum* were analyzed and sorted by On-chip Sort. Histograms describe the distributions of the fluorescence intensities of the droplets (I) immediately after droplet generation and (J) after 6 days of incubation. Microscopic images in (K) bright-field and (L) fluorescence mode show the sorted droplets with fluorescence intensities above the red threshold line in S1J Fig.
(TIF)

S2 Fig Sorting of droplets containing bacteria from soil. Approximately 100,000 droplets were analyzed per run using On-chip Sort. A total of 4 runs were performed on days 0 and 1, and a total of 3 runs were performed on days 3 and 7. One histogram from each day was selected and is shown in Fig 5, while the remaining are shown here. Histograms show the distributions of droplet fluorescence intensities after (A) 0, (B) 1, (C) 3, and (D) 7 days of cultivation. The red lines represent the sorting threshold. Each inset shows a histogram of fluorescence intensity above (B) 400, (C) 600, and (D) 800.
(TIF)

S3 Fig Observation of droplets containing bacteria from soil. (A) Dark-field and (B) fluorescence micrographs showing droplets containing soil bacteria and FRET-based RNA probe after 7 days of incubation. (C) Histogram describing the distributions of droplet fluorescence intensities after 7 days of incubation. The red line represents the sorting threshold. (D) Dark-field and (E) fluorescence micrographs showing the sorted droplets with fluorescence intensities above the red threshold line.
(TIF)

S1 Table Quantitative analysis of sorting droplets with growing model bacteria. ^a^ The number of droplets with fluorescence intensities above the threshold (red lines in Fig 4B and S1B, S1F and S1J Fig). ^b^ The percentage of droplets with fluorescence intensities above the threshold (red lines in Fig 4B and S1B, S1F and S1J Fig).
(XLSX)

S2 Table CFUs per droplet of model bacteria cultivated in droplets. (XLSX)

S3 Table The number of raw, chimera-removed, and effective reads and OTUs. ^a^ S-D0: Soil; SS-D0: Supernatant from soil after sonication and centrifugation; SD-D1, SD-D3, and SD-D7: Droplets sorted on days 1, 3, and 7, respectively; UD-D7: Droplets unsorted on day 7; BC-1, BC-D3, and BC-D7: Bulk cultures sampled on days 1, 3, and 7, respectively.
(XLSX)
