# Supplementary figures and images for: Fluorescent nucleic acid probe in droplets for bacterial sorting (FNAP-sort) as a high-throughput screening method for environmental bacteria with various growth rates
Source: PLoS One. 2019 Apr 17;14(4):e0214533. doi: 10.1371/journal.pone.0214533 (PMC6469844; doi:10.1371/journal.pone.0214533)

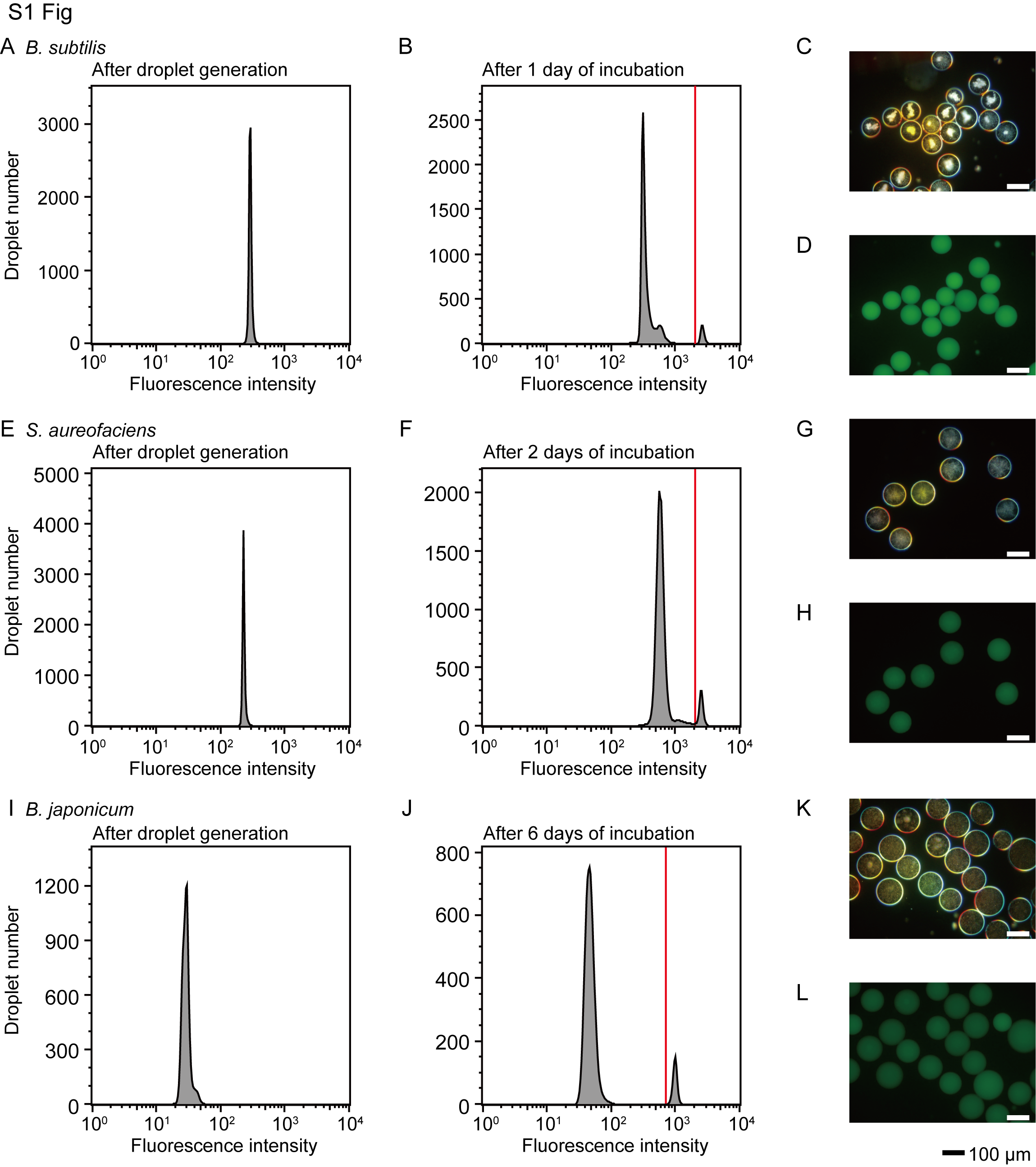

Supplement: S1 Fig — (A–D) Droplets with B. subtilis were analyzed and sorted by On-chip Sort. Histograms describe the distributions of the fluorescence intensities of the droplets (A) immediately after droplet generation and (B) after 1 day of incubation. Microscopic images in (C) bright-field and (D) fluorescence mode show the sorted droplets with fluorescence intensities above the red threshold line in S1B Fig. (E–H) Droplets with S. aureofaciens were analyzed and sorted by On-chip Sort. Histograms describe the distributions of the fluorescence intensities of the droplets (E) immediately after droplet generation and (F) after 2 days of incubation. Microscopic images in (G) bright-field and (H) fluorescence mode show the sorted droplets with fluorescence intensities above the red threshold line in S1F Fig. (I–L) Droplets with B. japonicum were analyzed and sorted by On-chip Sort. Histograms describe the distributions of the fluorescence intensities of the droplets (I) immediately after droplet generation and (J) after 6 days of incubation. Microscopic images in (K) bright-field and (L) fluorescence mode show the sorted droplets with fluorescence intensities above the red threshold line in S1J Fig. (TIF) [file pone.0214533.s001.tif]

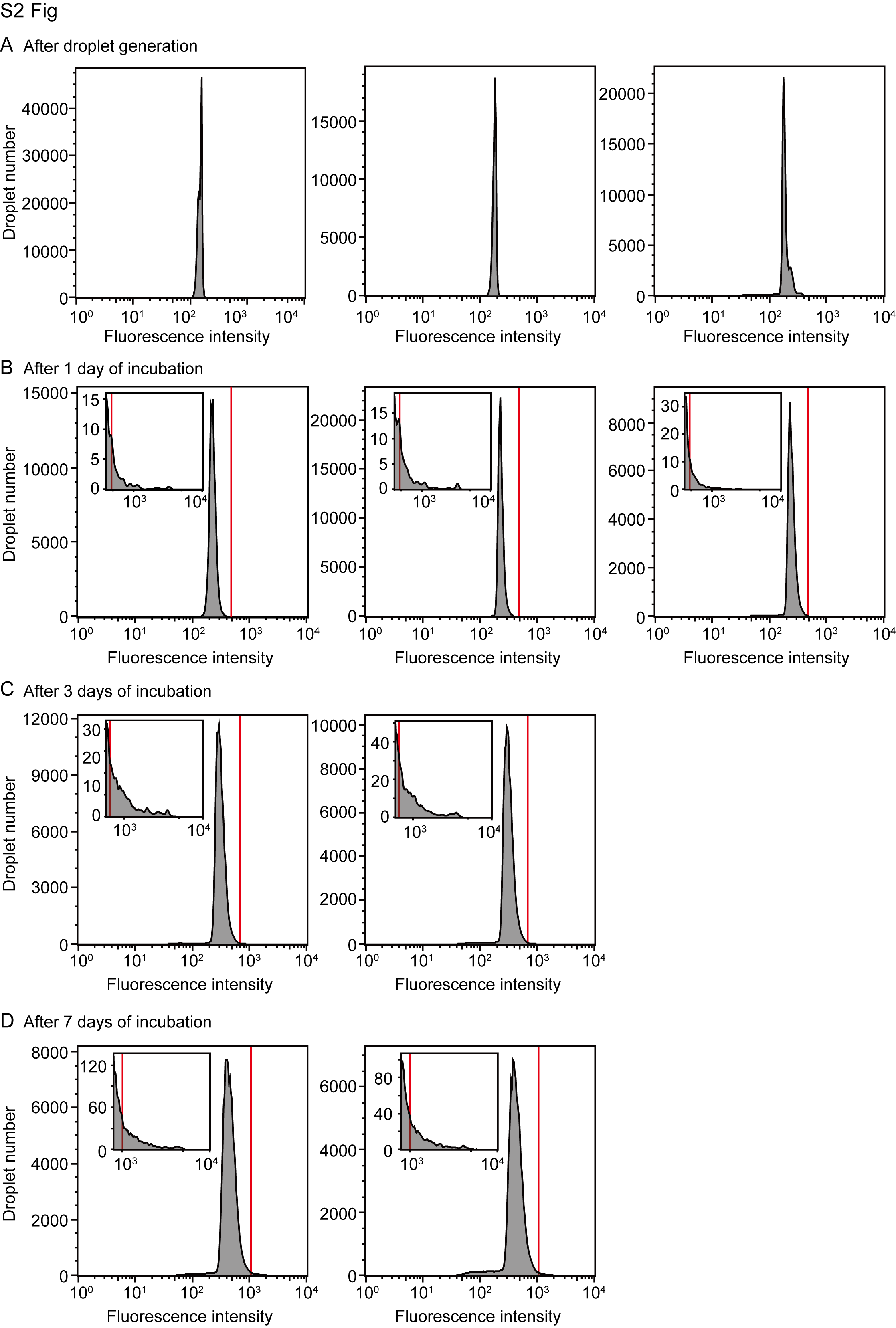

Supplement: S2 Fig — Approximately 100,000 droplets were analyzed per run using On-chip Sort. A total of 4 runs were performed on days 0 and 1, and a total of 3 runs were performed on days 3 and 7. One histogram from each day was selected and is shown in Fig 5, while the remaining are shown here. Histograms show the distributions of droplet fluorescence intensities after (A) 0, (B) 1, (C) 3, and (D) 7 days of cultivation. The red lines represent the sorting threshold. Each inset shows a histogram of fluorescence intensity above (B) 400, (C) 600, and (D) 800. (TIF) [file pone.0214533.s002.tif]

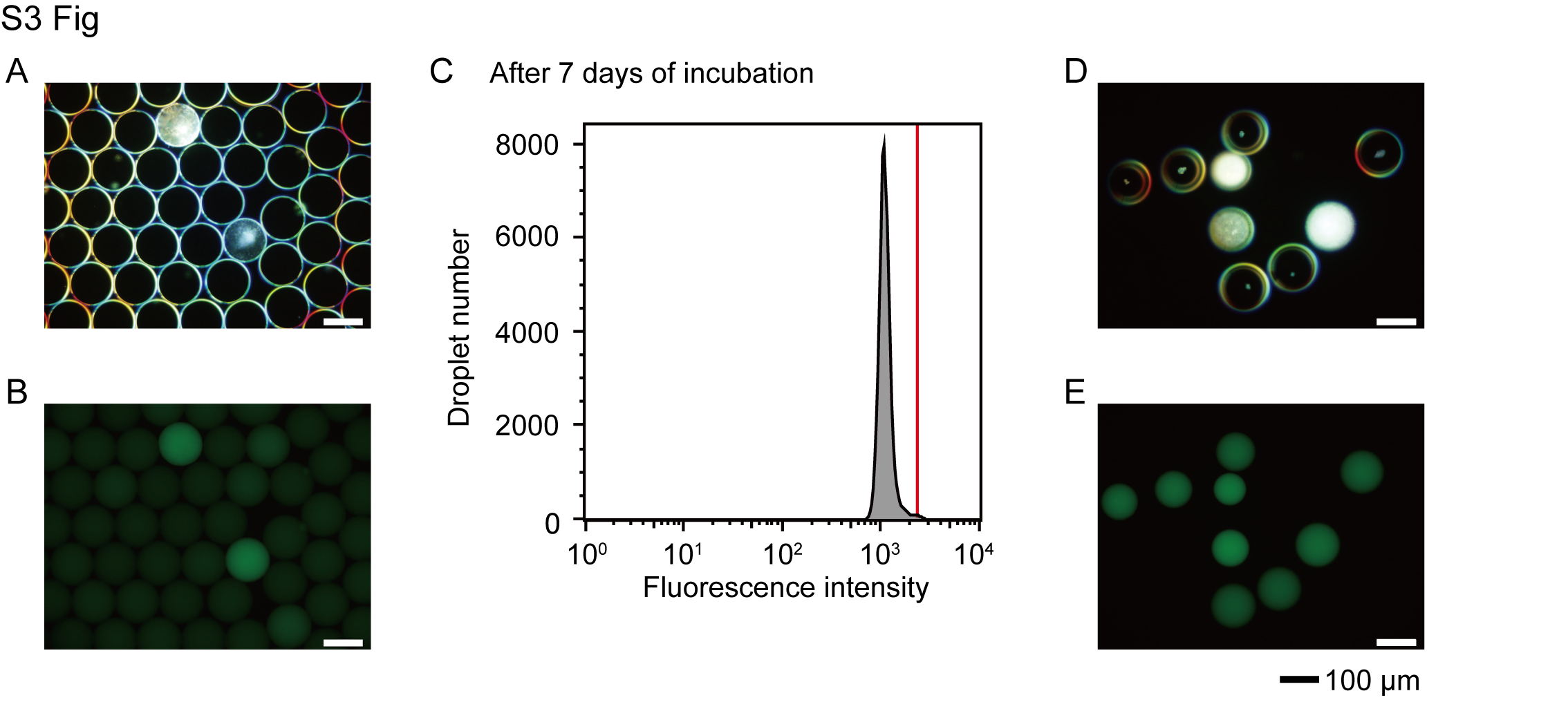

Supplement: S3 Fig — (A) Dark-field and (B) fluorescence micrographs showing droplets containing soil bacteria and FRET-based RNA probe after 7 days of incubation. (C) Histogram describing the distributions of droplet fluorescence intensities after 7 days of incubation. The red line represents the sorting threshold. (D) Dark-field and (E) fluorescence micrographs showing the sorted droplets with fluorescence intensities above the red threshold line. (TIF) [file pone.0214533.s003.tif]
